# Analysis of Hazardous Elements in Children Toys: Multi-Elemental Determination by Chromatography and Spectrometry Methods

**DOI:** 10.3390/molecules23113017

**Published:** 2018-11-19

**Authors:** Katarzyna Karaś, Marcin Frankowski

**Affiliations:** Department of Water and Soil Analysis, Faculty of Chemistry, Adam Mickiewicz University in Poznań, Umultowska 89 b, 61-614 Poznań, Poland; katarzyna.karas@amu.edu.pl

**Keywords:** chromium speciation, hazardous elements, toys safety, migration, ICP-MS

## Abstract

This paper presents the results of determination of hazardous metal (Cd, Cu, Cr, Hg, Mn, Ni, Pb, Zn) and metalloid (As, Sb) levels in toys available in the Polish market. Two independent sample preparation methods were used to determine the concentration and content of the metals and metalloids. The first one is defined by the guidelines of the EN-71 standard and undertook extraction in 0.07 mol/L HCl. This method was used to conduct speciation analysis of Cr(III) and Cr(VI), as well as for the determination of selected metals and metalloids. The second method conducted mineralization in a HNO_3_ and H_2_O_2_ mixture using microwave energy to determine the content of metals and metalloids. Determination of chromium forms was made using the high-performance liquid chromatography inductively coupled plasma mass spectrometry (HPLC-ICP-MS) method, while those of metals and metalloids were made using the ICP-MS technique. Additionally, in order to determine total content of chromium in toys, an energy dispersive X-ray fluorescence spectrometer (EDX) was used. The results of the analyses showed that Cr(VI) was not detected in the toys. In general, the content of heavy metals and metalloids in the studied samples was below the migration limit set by the norm EN-71.

## 1. Introduction

The amount of heavy metals in the environment is still increasing mainly due to anthropogenic activities that include industrial processes, power engineering, communication development, and the use of fertilizers and pesticides [1]. News about contamination of heavy metals in toy materials continues to be alarming. Some of them like arsenic (As), chromium (Cr), mercury (Hg), lead (Pb), selenium (Se), and zinc (Zn) are commonly used as coloring agents and catalysts to provide desired softness, brightness, and flexibility [2,3,4,5,6]. The maximum levels of metal contamination should be strictly regulated and kept at the lowest level that are technically feasible or are of no toxicological concern because of the toxicity and effects of bioaccumulation. It causes a variety of diseases, disorders, impairments, and organ malfunctions. This is important especially for children because they are considered more susceptible to hazardous metal substances compared to adults; they have higher basal metabolic rates, higher comparative uptakes of food, and lower toxins elimination rates. Moreover, children’s organs or tissues are developing, and thus, more sensitive to perturbed cellular processes [7,8,9]. Children typically spend a large amount of time playing with toys; toxic chemicals can be transferred from contaminated surfaces or soil to the hand and then ingested via hand-to-mouth activity. Threat of ingestion is common with children especially during the oral stage, which spans from birth until the age of 6 [10,11,12,13]. For children less than six years old, object mouthing is a common behavior and mouthing frequency and duration are especially high for infants (6–12 months) and toddlers (1–3 years). The knowledge of the total metal concentration in a solid sample is not sufficient to predict metal reactivity and effects on human health; indeed, metals can be present in various forms with different chemical properties and consequently different potential toxicity for humans [14]. One of the important elements is chromium, which has various oxidation states from II to VI [15]. Each of the forms presents different chemical properties and toxicity. The two most widespread forms of chromium in the environment are Cr(III) and Cr(VI). The intermediate states Cr(II), Cr(IV), and Cr(V) are unstable products in oxidation and reduction reactions of trivalent and hexavalent chromium, respectively. In various physiochemical processes, the different forms of chromium might undergo specific transformations, changing from one into another [16]. The inorganic trivalent form of chromium (Cr(III)) is relatively nontoxic and is an essential element in mammalian diets, especially in human beings [17,18]. It is a necessary microelement, which is involved in carbohydrate, lipid, and protein metabolism. By contrast, at the cellular level, hexavalent chromium (Cr(VI)) is a highly active carcinogen [19,20]. It can penetrate biological membranes and is recognized as a toxic substance. The ability to diffuse through the cell membranes is possible due to structural similarity of CrO_4_^2−^ ion to anions as SO_4_^2−^ or PO_4_^3−^, which are transported by their respective ion exchange channels [16,21]. Cr(VI) could reduce the amount of mitochondrial DNA (mtDNA) and inhibit mitochondrial electron transport chain complex I, resulting in perturbation of mitochondrial respiration and redox homeostasis. Human oral exposure to Cr(VI) produces hepatotoxicity and there is evidence that Cr(VI) accumulation occurs by oral exposure route mostly in the liver, which is the largest detoxification organ. Moreover, Cr(VI) may cause primary liver cancer and increase the risk of deterioration of cancer patients [7]. In Europe, toys must meet the criteria and requirements set by the European Commission Toys Safety Directive to carry the CE (Conformité Européenne) mark. All European Union member states have transposed this directive into law. The presence of Cr(VI) in toys sold in the European Union (EU) is strictly limited by the Toy Safety Directive (2009/48/EC) which ensures the safety of children by minimizing their exposure to potentially hazardous or toxic toy products, bearing in mind the young children’s tendency to mouth objects [22,23,24,25]. Part 3 of the standard—EN71-3+A1:2014-12—entitled “Migration of certain elements” subsequently outlines the migration limits of 18 elements from various categories of toy products, to enable the testing of toys for its compliance with legal provisions [24,25,26,27]. Toy materials are divided into three categories:

I: Dry, brittle powder-like or pliable materials;
II: Liquid or sticky materials;
III: Coatings and scraped-off materials.

Chromium has separate migration limits for Cr(III) and Cr(VI), which are 9.4 and 0.005 mg/kg, respectively [23]. Different analytical systems have been used for speciation analysis. The first group consists of methods requiring pretreatment, which are UV–visible spectrophotometry, atomic absorption spectrometry (AAS), inductively coupled plasma optical emission spectrometry (ICP-OES), and inductively coupled plasma mass spectrometry (ICP-MS). The other methods consists of hyphenated techniques such as flow injection analyzer (FIA), coupling of liquid chromatography with inductively coupled plasma mass spectrometry (LC-ICP-MS) with different separation modes, and capillary electrophoresis (CE). The second group involves solid techniques such as X-ray photoelectron spectroscopy (XPS), X-ray absorption near-edge spectroscopy (XANES), X-ray diffraction (XRD), and low-energy-electron-induced X-ray spectroscopy (LLEIXS) [15]. As the chromium species content in such samples is rather low, the coupling of liquid chromatography with inductively coupled plasma mass spectrometry (HPLC–ICP-MS) is a most often used technique, which is based on the combination of a separation method with an element-selective detection system [28]. Anion exchange columns and columns, which have both anion and cation exchange capabilities, are especially used for chromium speciation analysis [29]. High Performance Liquid Chromatography Inductively Coupled Plasma Mass Spectrometry (HPLC-ICP-MS) is often used in analyses of different environmental matrices such as natural water, soils, sediments, etc. [30]. In this study, for the first time, this technique was used to analyze samples of toys. The choice of this method is also justified by the fact that the ICP-MS analytical technique is one of the most sensitive and robust techniques, which offers pronounced advantages for its elemental specificity, wide linear dynamic range, and significantly low detection limits [31]. This method is also one of the most powerful analytical techniques for the collection of elemental information, offering LODs in the low- to sub-ng/L range for most elements. A wide linear dynamic range, multielement capabilities, survivable spectra, and the possibility of high sample throughput further characterizes this technique [30].

The objectives of this study are: (1) application of energy dispersive X-ray fluorescence spectrometry to determine the total content of chromium in toys; (2) development of a method for speciation analysis of chromium (III and VI) in toys; (3) determination of chromium, metals (Cd, Cu, Hg, Mn, Ni, Pb, Zn), and metalloids (As, Sb) by ICP-MS in HCl extracts and after microwave-assisted mineralization in HNO_3_/H_2_O_2_ mixture; and (4) assess the safety of toys particularly intended for younger children based on the requirements contained in EN71-3 norm Part 3: Migration of certain elements.

## 2. Results and Discussion

### 2.1. Application of Energy Dispersive X-ray Fluorescence Spectrometry to Determine Total Content of Chromium in Toys

Despite the use of nondestructive methods that did not require any sample preparation and measuring the toy material in different places and pieces several times, the results from the analysis showed that chromium was not detected in any toy material in mg/kg levels. But the fact that the chromium content was not found at such a level does not mean that it does not exist at all. The need to confirm this assumption was the starting point for the speciation analysis of chromium.

### 2.2. Method Development for Speciation Analysis of Chromium

**Figure 1:**
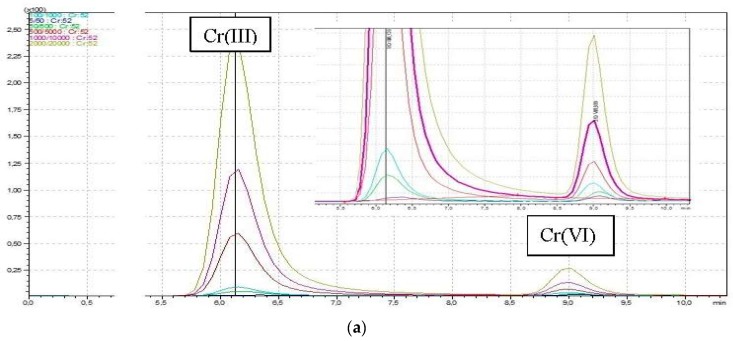

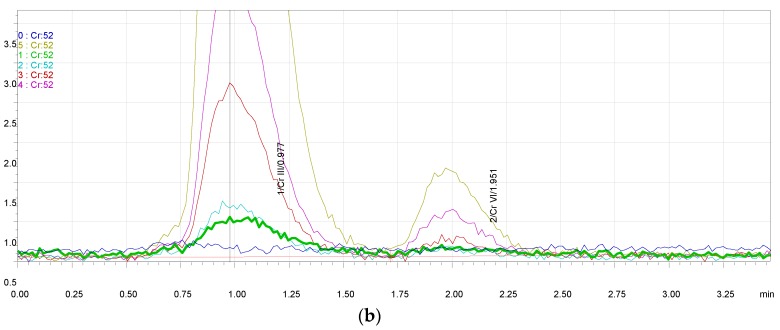
(**a**) Overlaid chromatograms for HPLC-ICP-MS with the use of Hamilton PRPX-100 analytical column. (**b**) Overlaid chromatograms for HPLC-ICP-MS with the use of Bio Wax analytical column.

A series of standard solutions containing 5, 10, 20, 50, and 100 ng/L Cr(VI), and 10 times higher concentrations of Cr(III) were prepared. The Cr species were completely resolved on anion exchange Hamilton PRPX-100 250 mm × 4.1 mm (10 µm) column with retention times of 6.1 min for Cr(III) and 9.1 min for Cr(VI). Because of the column length and extended retention time, a different Bio WAX nonporous 50 mm × 4.6 mm (5 µm) column was used. The Cr species were completely resolved with retention times of 0.97 min for Cr(III) and 1.98 min for Cr(VI). For both methods, good separation of standard solutions and of Cr(III) and Cr(VI) was obtained. Figure 1a,b presents the overlaid chromatograms for HPLC-ICP-MS.

Because of their shorter retention times for chromium, the Bio WAX column was selected for testing real samples. The LOD values for Cr(III) were equal to 1.3 ng/g in solid sample and 2.6 ng/L in solution, and for Cr(VI), 0.7 ng/g in solid sample and 1.4 ng/L in solution. However, in order to avoid tailing the peak connected with the higher concentration of Cr(III) in toys, higher concentrations of mix standard solutions containing: 10,000 ng/L Cr(III) and 1000 ng/L Cr(VI) were prepared and analyzed. In order to maintain high concentrations of Cr(III), high concentrations of Cr(VI) were also maintained, therefore peak resolution was satisfied.

### 2.3. Method Application for Toys’ Analysis

The newly developed method was applied to determine the concentration of Cr(III and VI) in toy samples collected from several shops in Poznań city (Poland). Table 1 shows the results for samples divided into individual colors and classified to two categories: cheap and expensive.

Concentration of Cr(VI) was under LOD in each analyzed samples. This is probably because in case of toys samples, as solid samples, the extraction process was applied. The acid used in the analysis was diluted 0.07 mol/L hydrochloric acid, and this concentration was in line with the requirements of the EN-71 norm. Such a low concentration was aimed simulating natural conditions, which could occur if a child swallowed part of a toy. This low concentration of HCl was too low to extract the analyzed form of chromium, which could have been retained in the toy material. Among the collected samples, the least numbers were brown and violet—two samples each. The average concentration of chromium(III) in brown samples was 108.8 ng/g and 187.0 ng/g in violet samples. Also, a small group was made up of metallic samples in which the concentration of trivalent form of chromium was significantly higher as expected than in other samples, and simultaneously was the one which exceeded migration limits observed in the whole analysis. The average concentration of chromium(III) in metallic samples was 2240 ng/g. One group of the collected samples were blue—15 samples, green—15 samples, and yellow—20 samples. The average concentration of this form of chromium was 206.9 ng/g in blue samples, 464.6 ng/g in green samples, and 276.7 in yellow samples. The maximum concentration of chromium(III) was observed in green sample and was equal to 2317 ng/g and in yellow samples was equal 1096 ng/g. The minimum concentration of chromium(III) was noticed in yellow samples and equaled 39.1 ng/g. Another group of samples consisted of ten samples each of pink and red. The average value of concentration of the detected form of chromium was 251.6 ng/g for pink samples and 219.8 ng/g for red samples. The minimum concentration of trivalent form of chromium was 87.6 ng/g in pink samples and 51.4 ng/g in red samples. However, the maximum was 397.2 ng/g in pink samples and 375.5 in red samples. The next group were orange, transparent, and white samples—six samples each. The average concentration of chromium(III) was 227.8 ng/g in orange samples, 154.3 ng/g in transparent samples, and 222.8 ng/g in white samples. The minimum concentration of chromium(III) in orange samples was 199.5 ng/g, in transparent samples was 102.4 ng/g, and in white samples was 52.9 ng/g. The maximum was 285 ng/g in orange samples, 282.3 ng/g in transparent samples, and 443 ng/g in white samples. Higher concentrations of chromium(III) were observed in black, green, and yellow parts of the toys, which can be connected with the pigments and paints usually used in toy production processes.

### 2.4. Determination of Chromium, Metals, and Metalloid Concentrations in HCl Extracts by ICP-MS

In order to compare the results from speciation analysis, the concentration of chromium in the extracts was measured by ICP-MS. The obtained results between both of the methods were comparable and the differences between the results were under 5%, which indicates the correctness of the results. High concentrations of chromium were noted for black, green, and yellow samples, the same as in speciation analysis. Reviewing the data obtained by analysis of HCl extracts, which are presented in Table 2, it may be stated that the higher concentration of element in black, brown, green, red, transparent, white, yellow, and metallic samples was noted for zinc.

The higher value of concentration of zinc was observed in green samples and was equal to 3066 ng/g. For blue and violet samples, the higher concentration was noted for copper and was equal to 1163 ng/g for blue color and 776 ng/g for violet color. For the last group of samples, orange and pink, the higher concentration was observed for nickel, which was equal to 421.5 ng/g for orange samples and 1024 ng/g for pink samples.

### 2.5. Determination of Chromium, Metals, and Metalloids Concentrations in HCl Extracts by ICP-MS Versus Total Content after Mineralization in HNO_3_/H_2_O_2_ Mixture

To complete the research, the determination of total content of chromium, metals (Cd, Cu, Hg, Mn, Ni, Pb, Zn), and metalloids (As, Sb) after microwave-assisted mineralization in HNO_3_/H_2_O_2_ mixture were made. The obtained values of concentration, which are much higher than that of HCl extracts, are related to the much greater elution strength of the HNO_3_/H_2_O_2_ mixture compared to the migration solution used in the procedure described in the EN-71 standard involving the use of HCl. This would mean that the metals and metalloids, which are contained in the toy material, are deeply bonded with this material and do not pass into the diluted HCl environment after swallowing the piece of the toy. These elements can be washed out only after using a stronger reagent such as a mixture of HNO_3_/H_2_O_2._ Differences in efficiency of extraction of the metals and metalloids were dependent on some factors like the type of metal, color of sample, and variety of toy material. Based on this result, it may be stated that the degree of metal washing out of the toy material using dilute 0.07 mol/L HCl was the highest for As, Cd, and Cu in pink samples; Cr, Hg, and Ni in black samples; Mn and Sb in red samples; Pb in brown samples; and Zn in violet samples. While these results after mineralization process indicate that the degree of metal washing out of the toy material was the highest for As, Cd, Cr, Ni, and Pb for metallic samples; Cu in blue samples; Hg in pink and white samples; Mn in metallic and black samples; Sb in metallic and blue samples, and Zn in brown samples. Procedures using a mixture of HNO_3_/H_2_O_2_ allowed determination of total content of chosen metals and metalloids. The obtained average content of elements in various colors of toys after ICP-MS analysis are presented in Table 3.

The obtained results are very similar to HCl extracts, the highest content of was very much present in the parts of toys made from metallic materials. The highest obtained values relate to As, Mn, and also Ni. In the case of chromium, the higher content was observed in green and yellow parts of the toys, also similar to speciation and HCl extract analyses. The highest concentrations of zinc were noted for black, brown, green, orange, pink, red, transparent, white, and yellow samples. The highest concentration was observed in yellow samples and was equal to 46.21 ng/g (excluding metallic samples). Higher concentration of copper was noted in blue samples, similar to HCl extract analysis. In violet samples, the higher concentration was noted for lead and was equal 722.8 ng/g. Presence of metals like Cr, Cd, Mn, Pb, and Zn in toy material may be due to pigments or coloring agents, which are usually added to toy materials during the production process. In addition, Mn is usually used as main additives in paints and Pb as stabilizer to improve material properties and reduce cost on plastic. Both Cd and Hg are also used as a stabilizing factor for PVC materials. Chromium is an important dye used in PVC materials and as a heat stabilizer [32]. As mentioned, the higher content of chromium was noted in black parts of toys. Black spinel-type chromium-nickel pigment is inert with respect to alkali and acids and has good mechanical strength [33]. Higher content of chromium was found also in yellow parts of the toys, which can be associated with yellow paints containing common pigment, e.g., lead chromate [8]. Synthetically produced chrome is using in paints and as material: vinyl, rubber, and paper. Higher content of chromium was noted also in green parts of the toys. Green chromium(III) oxide is commonly used in the paint industry. In particular, it is used to color paints, plastic, construction materials, refractories enamels, etc. [34]. Results obtained are similar to previous reports employing other analytical techniques such as ICP-OES and ED-XRF; XRF was used to determine migration limits of elements from different parts of the toy. Concentration of elements in plastic parts of the toys was below the specified limits stated in the European Union Toy Safety Directive [4,10]. Similar to studies on toy jewelry, metallic parts of the toys are more problematic than other materials [12,13]. In particular, special attention should be payed to the risk for children due to possible exceedance of migration limits. Similar to research conducted by high definition X-ray fluorescence techniques, in most of the samples of this study high amounts of Zn and Cu and other elements such as As, Cr, Mn, and Ni in smaller quantities were detected [2,6]. The smallest quantity was observed for Hg in a previous study, which investigated heavy metal concentrations in toys purchased from the Palestinian Market. This can be associated with the usage of mercury as a catalyst in specific chemical reactions during plastic manufacturing in China [2]. Compared to previous legislation on potentially harmful chemicals in children’s products, the migration limits of elements are significantly lower [27,35]. Possible exceedances usually refer to cadmium and lead-like factors commonly used as a stabilizer to prevent creating free chlorine radicals in materials made of PVC and also to reduce the cost of plastic [6,8,36]. The differences in metal and metalloid content in different toys and its parts are related with specificity of their compositions and differences in manufacturing processes [9]. Compared to similar speciation analysis of chromium conducted on dairy and cereal food samples, the results agree with those of this study. Trivalent chromium in yogurt and cheese samples ranges approximately from <13 to 255 ng/g. Most studies suggested the absence of hexavalent chromium in food samples like dairy and cereal products [37], chocolates, beverages, vegetables, fruits, eggs, meat and sea products [38], and flour [39], similar to the results from toy sample analyses obtained in this study.

## 3. Materials and Methods

### 3.1. Sample Collection

**Figure 2:**
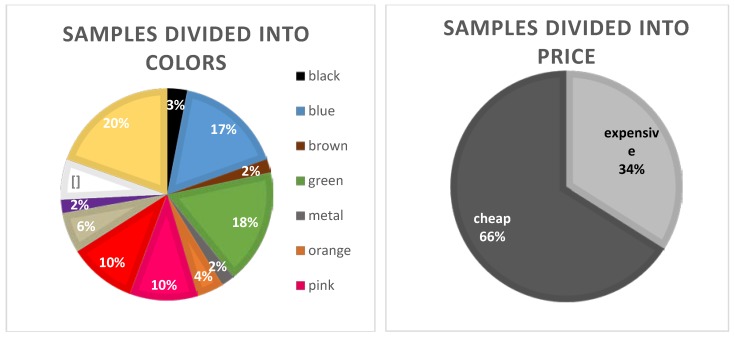
Samples divided into factors: colors and price.

Toys were collected randomly from several convenience shops in urban areas in Poznań (Poland). These were shops commonly visited by people, which offer different types of toys at various prices. The samples were segregated into parts with different colors (black 3%, blue 17%, brown 2%, green 18%, metal 2%, orange 4%, pink 10%, red 10%, transparent 6%, violet 2%, white 6%, and yellow 20%). In addition, to answer the question of whether more expensive toys are safer for the health of our children, a comparative analysis of the content of chromium in toys divided into cheap toys <5 € (34%) and expensive >5 € (66%) was made. The division is presented on Figure 2.

### 3.2. Determination of Total Chromium Content by Energy Dispersive X-ray Fluorescence Spectrometry

In order to determine the total content of chromium in toys, energy dispersive X-ray fluorescence spectrometer (EDX) was used. Each toy categorized based on color and price was irradiated with X-rays from an X-ray tube after selecting the most appropriate irradiation diameter for the sample shape. Due to the possible heterogeneity of the toy, each one was measured several times in different parts of the toy. During analysis, a special filter for chromium was used to improve the sensitivity of detection and reduce or eliminate factors such as background, characteristic lines, and other forms of scattered radiation. The analytical conditions for EDX is presented in table (Table 4).

### 3.3. Method Development for Speciation Analysis of Chromium(III and VI) in Toys

#### 3.3.1. Analytical System

An ICPMS-2030 mass spectrometer (Shimadzu, Japan) directly coupled with Prominence LC 20Ai inert system was used for Cr speciation. The inert system eliminates the possibility of the metal background leaching from the components of the aperture. In addition, inert LC is the most suitable for metal speciation analysis in which the lowest possible detection limit is required. The ICP-MS operates at 1000 W with 9 L/min Ar plasma gas flow, 1 L/min nebulizer Ar gas flow, and 0.75 L/min auxiliary Ar gas flow for Hamilton PRP X100 column and 0.70 L/min auxiliary Ar gas flow for BioWAX column. The concentric (MicroMist) nebulizer with 1.0 L/min (carrier + make-up) argon gas flow was used for nebulizing the HPLC eluate. The kinetic energy discrimination (KED) mode was used for determination of chromium isotope 52.

The sampling depth was 4.5 mm for Hamilton PRP X100 column and 5.0 mm for BioWAX column. The inert LC is equipped with a binary pump LC 20Ai, a vacuum degasser (DGU 20A3R), an autosampler (SIL 20AC), a heated column compartment (CTO 20AC), and a controller (CBM 20A)(Shimadzu, Kyoto, Japan). An anion exchange column, Agilent Bio WAX 5 μm 4.6 mm × 50 mm, 5 μm, PEEK guard (Agilent, Santa Clara, CA, USA), was used for resolving of Cr species at ambient temperature. Polypropylene vials fitted with polypropylene vial caps were used. Prior to use, the vials were cleaned with dilute nitric acid and thoroughly rinsed with ultrapure deionized water (UPW) (Merck, Kenilworth, NJ, USA). Rubber, plastic, and even trace organic residues can easily cause reduction of Cr species when the sample comes into contact with them. Liquid Chromatography parameters are presented in Table 5.

#### 3.3.2. Reagents and Standards

Ultrapure water (<0.005 µS) obtained from a Milli-Q Direct 8 purification unit (Millipore, Burlington, MA, USA, Merck) was used to prepare all the solutions. Ammonium nitrate, potassium dichromate, standard solution of Cr(III) and Cr(VI)—1000 mg/L (Merck, USA), Na-EDTA, diluted hydrochloric acid, and ammonia (Sigma−Aldrich, St. Louis, MI, USA) to adjust the pH of the mobile phase were used for the analysis. All standard solutions used for the calibration process were prepared by volume dilution of Cr standard solution 1000 mg/L. To avoid contamination, all glassware and storage bottles were kept in 10% (*v*/*v*) nitric acid for at least 48 h, rinsed three times with ultrapure water, and preserved dried till use.

#### 3.3.3. Sample Preparation

**Figure 3:**
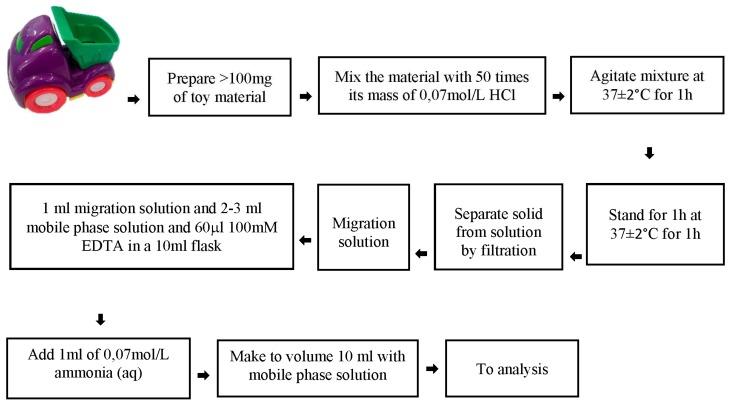
The sample preparation procedure for chromium speciation analysis.

The procedure used HPLC-ICP-MS and followed EN71-3, which simulated gastric digestion as would occur in the case when a child swallows toy material; this is presented in Figure 3.

The extraction (migration) solutions obtained were stabilized with EDTA and ammonia solution. The addition of ammonia to neutralize the solution preserves the chromium species extracted from toy materials for several hours with no species inter-conversion or loss by precipitation. Both Cr species were preserved for at least 24 h after the sample preparation if the solution was neutralized at pH = 7 ± 0.1. Calibration standards were also prepared by the same sample preparation method. The guidelines of the EN71-3 define conditions that may make it difficult to prepare a sample properly. The test portion of the sample should be not be less than 100 mg and shall have at least one dimension of approximately 6 mm. Therefore, in element analysis, it was impossible to use metal tools to fragment the samples. In this case, instead of a ball mill or metal scissors, a Teflon hammer and scissors were used.

### 3.4. Determination of Concentration of Chromium, Metals, and Metalloids in HCl Extracts by ICP-MS

In order to check the correlation between concentration of chromium and other elements: As, Cd, Cr, Cu, Hg, Mn, Ni, Pb, Sb, and Zn, ICP-MS analysis after extraction in 0.07 mol/L HCl was conducted. The sample preparation procedure was the same as in Figure 2. The results of chromium determination were compared with the data obtained from chromium speciation analysis.

### 3.5. Determination of Total Content of Chromium, Metals, and Metalloids after Microwave-Assisted Mineralization in HNO_3_/H_2_O_2_

Every sample segregated based on color and price was solubilized by closed-vessel microwave-assisted acid digestion in an Anton Paar Multiwave Pro microwave oven equipped with 8NXF 100 rotor. A sample mass 100±0.2 mg was directly weighted into the microwave oven polytetrafluorethylene (PTFE) vessels. The attempt to extract a solid sample into a solution using only HNO_3_ failed. Because of that, the digestion was continued further with 8 mL of high-purity concentrated nitric acid (HNO_3_, 65%; Merck, USA) and 2 mL of high-purity hydrogen peroxide (H_2_O_2_, 30% *v*/*v*; TraceSELECT, Fluka, Seelze, Germany). This step significantly improved efficiency of this process. Each sample was analyzed in triplicates. The total content of metals and metalloids was determined using ICP-MS technique. The data obtained were compared with the results from HCl extract analysis. The parameters of ICP-MS spectrometer for analysis of both type of extracts are presented in Table 6.

## 4. Conclusions

The EDX analytical technique for detection of chromium was conducted with special filters, which allowed analysis at the level of mg/kg and did not require sample preparation. Despite using special filters for chromium, improving the sensitivity of detection, and reducing or eliminating factors such as background, characteristic lines, and other forms of scattered radiation, and measuring the toy material several times from different parts of the toy, the obtained results showed the absence of chromium in examined toy materials in the abovementioned levels. A method for speciation analysis of chromium was developed. The HPLC-ICP-MS hyphenated technique used allowed to obtain better selectivity and sensitivity. The hexavalent form of chromium was not detected in toys which were tested, which can be attributed to low elution strength of dilute HCl used in the procedure described in the standard and retaining this form of chromium in the toy material. Based on the results, the higher concentration of chromium(III) is in black, green, and yellow samples, but in none of the cases, the migration limit for this form of element was exceeded. Determination of chromium, metal (Cd, Cu, Hg, Mn, Ni, Pb, Zn), and metalloid (As, Sb) concentrations in HCl extracts by ICP-MS and of total content after microwave-assisted mineralization in HNO_3_/H_2_O_2_ mixture were performed. The data obtained for HCl extracts were comparable with results from speciation analysis, which suggest correctness of the research. Simultaneously, it was observed that acid digestion had much higher elution strength and had significantly higher concentration values of selected metals, and thus, made it possible to determine the total content of elements. Obtained data also provides information about the potential risk of exposure by being released by saliva during chewing, sweating during skin contact, or gastric fluid after ingestion. Besides, the toy samples were bought only from Poznan city area, which may not represent the whole country; however, most of them are available in stores with the same range of products in various places in Poland or even in the whole world. Considering the division into the price of toys, no significant differences in the content of the investigated metals and metalloids were noticed. The contents of these metals were comparable with each other. Although the migration limits of certain elements from the toy’s material to the environment were not detected above recommended values in these studies, toy materials may still pose a possible risk for children by creating negative health effects, particularly the metallic parts of the toys. This is because of the variety of the materials and the heterogeneity of element composition in different parts of the toy. The second argument is the possibility of bioaccumulation of heavy metals and metalloids from toys, which can happen due to ingestion, inhalation, or dermal contact, and which can last through childhood during the long times spent on playing and educating with toys.

## Figures and Tables

**Table 1:** Concentrations values of chromium(III) for particular samples.

Sample	Cr(III) [ng/g]											
	Black	Blue	Brown	Green	Metal	Orange	Pink	Red	Transparent	Violet	White	Yellow
**cheap toys < 5 €**												
4	A	227.0 ± 3.8	A	A	A	A	A	A	A	A	A	A
7	A	A	A	357.5 ± 5.2	A	A	211.0 ± 3.1	A	A	A	A	258.0 ± 4.4
8	A	A	A	A	A	A	A	A	145.0 ± 2.1	A	A	A
9	A	A	A	315.5 ± 4.9	A	A	A	A	A	A	A	A
12	A	A	A	542.0 ± 10.6	A	261.0 ± 5.0	A	A	A	A	A	585.0 ± 12.0
15	A	A	A	A	A	A	A	375.5 ± 8.1	A	A	A	296.5 ± 7.2
16	A	A	A	A	A	A	A	A	A	A	A	554.0 ± 13.5
17	A	A	A	510.5 ± 14.7	A	A	A	A	A	A	A	84.5 ± 1.2
18	A	A	A	A	A	A	A	260.0 ± 6.8	A	A	A	A
19	A	A	A	A	A	A	A	A	A	A	291.5 ± 8.7	220.0 ± 4.1
20	A	A	A	2317 ± 60	A	A	A	A	A	A	A	A
24	A	131.0 ± 1.4	A	A	A	A	A	294.5 ± 5.5	A	A	A	584.0 ± 9.9
25	A	A	A	A	A	A	A	221.0 ± 1.9	A	108.5 ± 2.0	A	239.1 ± 5.4
26	A	A	A	A	A	285.0 ± 6.0	A	A	A	A	A	81 ± 1.1
27	A	127.5 ± 2.5	A	73.5 ± 1.3	A	A	A	56.2 ± 0.9	A	A	A	151.5 ± d2.6
29	A	A	A	A	A	A	A	A	115.5 ± 2.0	A	A	A
30	A	361.5 ± 7.0	A	A	A	199.5 ± 2.5	140.5 ± 2.2	A	A	A	A	A
32	A	124.5 ± 1.1	A	A	A	A	A	127.5 ± 3.1	A	A	52.9 ± 0.9	48.5 ± 1.0
1	A	A	A	218.5 ± 4.7	A	A	A	A	A	A	A	A
2	A	A	A	A	A	A	A	A	282.3 ± 5.6	A	A	A
5	A	A	A	A	A	168.8 ± 2.7	A	A	A	A	A	A
7	A	109.6 ± 1.9	A	A	A	A	A	A	A	A	A	A
8	A	A	A	A	A	A	118.5 ± 1.7	A	A	A	A	A
10	A	A	A	A	A	A	142.6 ± 2,6	A	102.4 ± 2.0	A	A	A
11	A	595.4 ± 10.2	A	A	A	A	A	A	A	A	A	A
13	A	A	A	A	A	A	A	A	A	A	A	116.4 ± 1.8
15	8025 ± 177	71.7 ± 1.2	65.5 ± 1.2	A	A	A	153.9 ± 2.2	A	A	A	A	39.1 ± 0.8
17a	70.5 ± 1.4	145.5 ± 3.1	A	795.5 ± 15.9	A	A	A	A	A	A	A	80.9 ± 1.6
18a	A	A	A	A	312.3 ± 8.1	A	A	237.4 ± 4.4	A	A	A	130.5 ± 2.9
19	A	A	A	106.2 ± 2.4	A	A	A	A	A	A	A	A
20	A	A	A	A	A	A	A	A	A	A	188.2 ± 3.8	A
21	A	197.6 ± 2.1	A	A	A	A	A	A	A	A	A	A
22a	A	A	A	A	A	A	87.6 ± 2.1	A	A	A	A	1096 ± 25.4
**expensive toys > 5 €**												
1	A	245.0 ± 5.0	A	A	A	A	381.0 ± 9.0	A	A	265.5 ± 6.3	A	A
2	A	A	A	342.0 ± 6.8	A	A	A	A	A	A	A	A
3	A	209.0 ± 4.2	A	A	A	A	A	A	A	A	A	A
5	A	A	A	A	A	A	A	A	A	A	245.0 ± 4.7	A
6	A	A	A	203.5 ± 5.0	A	A	A	A	A	A	A	A
10	A	A	152.0 ± 3.1	A	A	A	A	A	A	A	A	397.0 ± 9.4
11	A	A	A	A	A	A	173.5 ± 3.5	A	A	A	A	A
13	A	A	A	A	A	A	710.0 ± 18.2	A	A	A	A	A
14	A	266.5 ± 6.8	A	A	A	-	A	A	A	A	A	A
21	A	A	A	A	A	-	A	421.0 ± 10.2	A	A	A	A
22	A	A	A	A	A	-	A	A	A	A	443.0 ± 9.4	A
23	A	A	A	349.0 ± 5.1	A	-	A	A	A	A	A	A
28	A	A	A	A	4168 ± 985	248.5 ± 5.1	A	A	A	A	A	A
31	A	A	A	A	A	A	A	A	154.5 ± 2.0	A	A	A
33	A	96.5 ± 1.1	A	48.5 ± 0.8	A	A	A	A	A	A	A	A
3	A	194.8 ± 1.9	A	A	A	203.7 ± 4.0	A	A	A	A	A	A
6	A	A	A	A	A	A	A	154.0 ± 2.0	A	A	A	A
9	A	A	A	A	A	A	A	A	126.0 ± 1.9	A	A	137.9 ± 2.4
12	A	A	A	A	A	A	397.2 ± 8.0	A	A	A	A	A
14	59.8 ± 1.1	A	A	124.7 ± 2.9	A	A	A	51.35 ± 0.9	A	A	116.4 ± 2.0	365.5 ± 8.1
16	A	A	A	664.5 ± 11.3	A	A	A	A	A	A	A	68.81 ± 1.4
Average value	310.9 ± 59.8	206.9 ± 3.7	108.8 ± 2.2	464.6 ± 10.1	2240.2 ± 494.5	227.8 ± 2.55	251.6 ± 5.26	219.8 ± 2.43	154.3 ± 2.6	187 ± 3.65	222.8 ± 4.42	276.7 ± 1.56

A - absent color in the toy.

**Table 2:** Average concentration of metals and metalloids in various colors of toys in HCl extracts.

	As ng/g	Cd ng/g	Cr ng/g	Cu ng/g	Hg ng/g	Mn ng/g	Ni ng/g	Pb ng/g	Sb ng/g	Zn ng/g
Black	32.75 ± 167	1.8 ± 65	281.2 ± 163,9	44.8 ± 5161	4.133 ± 3.6	39.67 ± 182	163 ± 54.5	9.3 ± 815	46.32 ± 63.7	461.7 ± 309.5
Blue	10.8 ± 27.8	12.34 ± 51.5	213.3 ± 126.5	1163 ± 9150	0.944 ± 4.8	94.9 ± 153.5	14.5 ± 421.6	49.17 ± 699.3	43.66 ± 18.4	397.2 ± 328.4
Brown	7.6 ± 13.4	1.8 ± 70	104 ± 146	44.8± 538.9	3.15 ± 9.77	65.33 ± 135	14.5 ± 23.5	9.3 ± 464.1	21.48 ± 4.55	938.9 ± 385.5
Green	10.3 ± 90.5	24.74 ± 275	477.9 ± 194	864.7 ± 2900	0.838 ± 4.35	364.6 ± 1071	14.5 ± 411.7	138.1 ± 338.2	193.7 ± 583	3066 ± 8491
Orange	21 ± 103.6	22.1 ± 56	218.1 ± 115.5	1570 ± 3850	1.32 ± 2.35	337.8 ± 154.2	421.5 ± 171.1	168.7 ± 500	37.02 ± 30.05	387.2 ± 477.6
Pink	76.9 ± 1205	2.9 ± 54.5	245 ± 184	90 ± 313.9	5.115 ± 4.7	288 ± 1236	1024 ± 424.8	9.3 ± 430	28.05 ± 28.1	397.2 ± 423.5
Red	31.3 ± 321.4	14.85 ± 94	230.5 ± 209	760 ± 211.7	0.955 ± 2.75	46.73 ± 594	443.6 ± 255.5	104.7 ± 645	243.6 ± 455.5	1746 ± 885.4
Transparent	13.9 ± 41.98	29.1 ± 126	158.8 ± 185	1058 ± 2368	1.675 ± 3.23	501.8 ± 1804	402.4 ± 721.6	61.67 ± 430	35.95 ± 22.3	2548 ± 1542
Violet	26.9 ± 53.5	16.25 ± 57.5	197.5 ± 145	776 ± 65.5	2.15 ± 3.4	6.3 ± 70.5	14.5 ± 103.4	91.67 ± 440	91.03 ± 140	392.7 ± 76
White	8 ± 20.4	1.8 ± 55	233.1 ± 136	216.7 ± 606.1	0.259 ± 0.15	38.83 ± 189	14.5 ± 298	9.3 ± 435	184 ± 184.6	743.8 ± 617
Yellow	12.3 ± 26.1	1.289 ± 142.5	289.5 ± 142.5	276.3 ± 4224	0.782 ± 5.8	22.36 ± 224.5	355.8 ± 398.4	5.614 ± 460	54.23 ± 49.7	1443 ± 974
**µg/g**										
Metallic	0.298 ± 0.296	0.282 ± 2.95	9.175 ± 0.194	7.35 ± 3.3	0.055 ± 0.001	119.6 ± 257.5	150.5 ± 25.39	1.622 ± 0.455	0.279 ± 0.001	902.7 ± 7.009

**Table 3:** Average content of metals and metalloids after mineralization in HNO_3_/H_2_O_2._

	As ng/g	Cd ng/g	Cr ng/g	Hg ng/g	Mn ng/g	Ni ng/g	Pb ng/g	Sb ng/g	Zn µg/g	Cu µg/g
black	67.5 ± 105	345.4 ± 65	311.1 ± 116.5	28.2 ± 3.6	541.5 ± 1261	163.0 ± 54.5	2816 ± 3025	2838 ± 846.9	6.718 ± 18.98	3.862 ± 5.092
blue	151.9 ± 427.5	115.3 ± 51.5	359.1 ± 318.5	48.2 ± 51.6	816.4 ± 875.3	394.3 ± 2087	479.2 ± 435	218.4 ± 422.2	13.45 ± 1.851	181.7 ± 18.76
brown	32.5 ± 36	56.5 ± 70	823.1 ± 1438	40.8 ± 17.2	1966 ± 3634	1806 ± 2124	449.6 ± 1193	161.9 ± 283.2	19.52 ± 2.458	0.971 ± 5.389
green	135.3 ± 134.5	127.7 ± 275	552.8 ± 1204	68.8 ± 104.7	728.5 ± 996.8	390.3 ± 618.1	724.9 ± 1455	3806 ± 981.9	32.39 ± 65.35	15.93 ± 29.25
orange	187.7 ± 154.1	125.1 ± 56	485.8 ± 696.9	47.9 ± 60.8	231 ± 557.5	421.5 ± 932.8	511.7 ± 500	198.1 ± 305.7	29.34 ± 54.37	17.89 ± 3.096
pink	105 ± 125	105.9 ± 54.5	461.2 ± 862.8	412.5 ± 26.31	2780 ± 111.1	1024 ± 734.41	690.8 ± 430	300.4 ± 1477	23.01 ± 42.66	6.767 ± 2.4
red	229.8 ± 311.5	117.9 ± 94	357.7 ± 469.2	49.2 ± 86.8	214.3 ± 17.3	443.6 ± 200	488.2 ± 645	243.6 ± 180.4	40.80 ± 48.37	1.424 ± 2.65
transparent	169.9 ± 183.5	156.5 ± 126	451.3 ± 767.5	91.9 ± 77.9	944.7 ± 382.5	402.4 ± 1175	552.9 ± 430	185.1 ± 22.3	7.185 ± 7.787	5.307 ± 2.5
violet	226 ± 340	119.3 ± 57.5	429.2 ± 64.8	57.1 ± 56.7	170.9 ± 163.1	322.9 ± 241.1	722.8 ± 440	426.9 ± 140	0.393 ± 0.01	0.340 ± 2.45
white	31.12 ± 83.5	101.8 ± 55	422.6 ± 367.9	35.3 ± 13.3	371.3 ± 601.7	508.8 ± 992.7	560.2 ± 435	184.0 ± 184,6	28.78 ± 11.36	0.615 ± 2.35
yellow	140.6 ± 591.4	104.3 ± 56.5	479.7 ± 839.3	36.7 ± 19.2	228.6 ± 465	355.8 ± 529.8	508.0 ± 460	185.7 ± 409.7	46.21 ± 66.85	1.946 ± 2.5
**mg/g**										
metallic	4.729 ± 9.25	0.002 ± 0.003	0.102 ± 0.124	0.002 ± 0.002	96.79 ± 189.9	919.7 ± 1837	0.452 ± 0.0003	0.004 ± 0.04	3.725 ± 1.542	0.037 ± 0.173

**Table 4:** Energy dispersive X-ray (EDX) analytical conditions.

Analytical Conditions	
X-ray tube	Rh target
Filter	Filter #1 (for Cr)
Voltage	Cr:30 kV
Current	5.22–6.22 keV
Atmosphere	Air
Measurement Diameter	10 mm Φ
Measurement Time	100 s
Dead Time	30%

**Table 5:** Basic Liquid Chromatogrpahy parameters for speciation analysis.

Column	Hamilton PRP X100	BioWAX Non-Porous
**Mobile Phase**	60 mM NH_4_NO_3_, pH 7.0 ± 0.1 by NH_4_OH	75 mM NH_4_NO_3_, Ph = 7.1 ± 0.1 by NH_4_OH
**Mobile Phase Flow [mL/min]**	1.0	0.8
**Temperature**	30 °C	30 °C
**The Volume of the Dispensing Valve Loop [µL]**	350	200

**Table 6:** ICP-MS parameters for analysis of HCl and H_2_O_2_/HNO_3_ extracts.

**Generator Power [W]**	1200
Argon flow—plasma [L/min]	8.0
Argon flow—nebulizer [L/min]	1.1
Argon flow—auxiliary gas [L/min]	0.70
Nebulizer	Concentric type, “micro”
Torch	Concentric type, “mini”
Spray chamber temperature [°C]	5.0
Collision gas—He [ml/min]	6.0
Voltage on octapole rods [V]	−21
Energy filter [V]	7.0
Sampling deep [mm]	5.0
